# An Atypical Presentation of Right Atrial Flutter Following the Cox Maze Procedure and Left Atrial Reduction Surgery: A Case Report

**DOI:** 10.19102/icrm.2017.080703

**Published:** 2017-07-15

**Authors:** Preet Cheema, Christian Perzanowski

**Affiliations:** ^1^Brandon Regional Hospital, Riverview, FL; ^2^Tampa General Hospital, Tampa, FL; ^3^Bay Area Cardiology, Brandon, FL

**Keywords:** Atrial flutter, atrial reduction surgery, Cox maze procedure

## Abstract

The onset of recurrent atrial tachyarrhythmia (ATA) following the Cox maze procedure (CMP) is commonly encountered, and may be associated with increased perioperative mortality. The majority of recurrent ATA cases are localized to the left atrium following surgical ablation. Right atrial flutter (AFL) following the CMP is a less-frequent occurrence, and may pose a diagnostic challenge due to uncharacteristic surface electrocardiogram (ECG) and intracardiac activation patterns. In this case, a 68-year-old male who had previously undergone left-sided surgical ablation with left atrial reduction for the treatment of persistent atrial fibrillation during coronary artery bypass and mitral valve repair developed symptomatic atypical AFL. The patient was intolerant to amiodarone, and was thus scheduled for ablation. Given the patient’s history of extensive left atrial instrumentation and surface ECG findings indicating an atypical AFL, the decision was made to proceed with left atrial mapping. During the electrophysiology study, initial activation mapping was not suggestive of a cavotricuspid isthmus reentrant arrhythmia. Here, we describe the possible mapping pitfalls associated with a persistent tachyarrhythmia that was ultimately proven to be a right AFL, despite atypical activation patterns.

## Introduction

The Society of Thoracic Surgeons recommends the Cox maze procedure (CMP) for the treatment of persistent atrial fibrillation (AF) in those patients who are undergoing cardiac surgery procedures, such as coronary artery bypass grafting or mitral valve repair. According to the current literature, the procedure has been proven to be successful in restoring sinus rhythm in symptomatic patients with persistent and permanent AF. Despite positive outcomes in the majority of patients, however, recurrent atrial tachyarrhythmias (ATAs) are commonly encountered following CMP, and are associated with an increased risk of perioperative mortality.^1^ Often refractory to conservative management, these recurrent arrhythmias are evasive and difficult to diagnose.

The most common recurrent ATA after CMP is AF, with the majority of cases initiating near the pulmonary veins or involving the mitral annulus and remaining localized to the left atrium.^2–5^ Right atrial flutter (AFL) is uncommon after CMP and may be challenging to diagnose electrocardiographically in patients who have undergone the procedure. The following case illustrates the importance of considering typical right atrial isthmus-dependent flutter, even when the electocardiographic and intracardiac activation patterns suggest otherwise.

### Case presentation

A 68-year-old male had undergone coronary artery bypass graft with mitral valve repair and concomitant CMP for persistent AF. At the time of surgery, the patient had a left atrial size of 6.9 cm in diameter, worsening left ventricular function, and congestive heart failure (CHF). The CMP lesion set included pulmonary vein isolation using surgical incision and bipolar radiofrequency to create a box lesion, and left atrial appendage ligation. Surgically, left atrial reduction was performed, reducing the atrial size from 6.9 cm to 5.4 cm postoperatively.

At the patient’s three-month follow-up appointment, it was observed that he had reverted back to displaying an atypical-in-appearance AFL. At this time, he reported symptoms of excessive fatigue and reduced exercise tolerance. The patient did not tolerate amiodarone due to the presence of bradycardia. The decision was made to proceed with percutaneous ablation to restore sinus rhythm.

**Figure 1:**
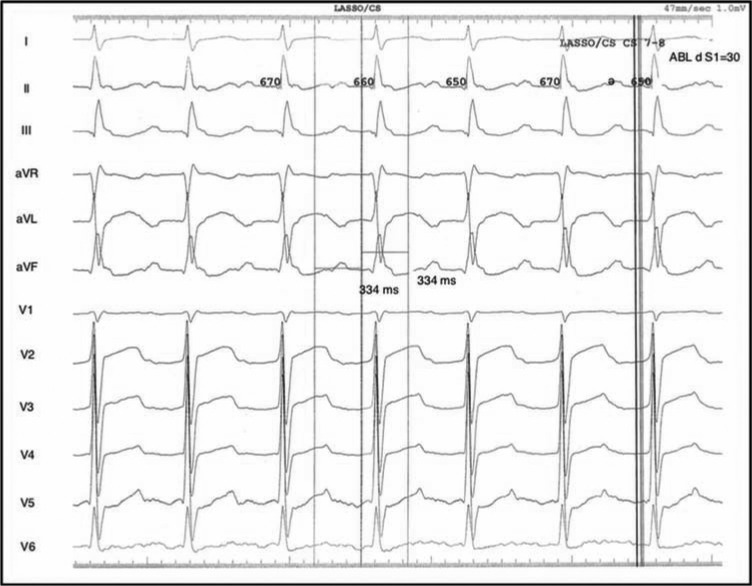
Presenting 12-lead electrocardiogram demonstrating organized atrial activation characteristic of an atypical flutter with 2:1 conduction. Note the broad flutter waves in this patient with extensive left atrial surgical intervention. The calipers reflect intracardiac cycle length measurement based on local electrograms.

**Figure 2:**
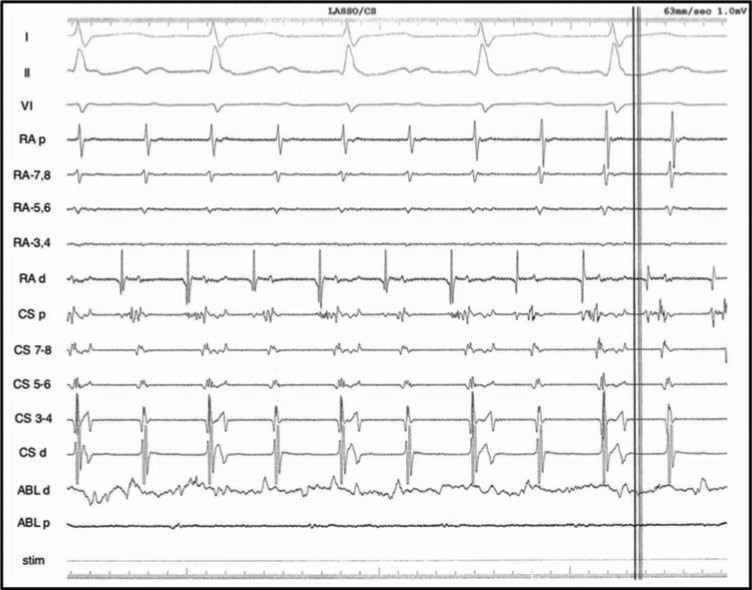
Baseline intracardiac recordings of the presenting arrhythmia. Note the fractionated activity in three of the proximal coronary sinus electrograms, which might be due to the prior left atrial debulking.

On initial assessment, the patient’s surface ECG displayed a left AFL pattern: specifically, positive P-waves in V1 and negative P-waves in leads AVL and I, with a 2:1 conduction and a cycle length of 334 ms **(Figure 1)**. During the electrophysiology study, the atrial activation pattern was not typical of a cavotricuspid isthmus (CTI)-dependent mechanism, and mitral isthmus tachycardia was subsequently suspected **(Figure 2)**. Given the proclivity for atypical flutters and ATA to degenerate into other atrial arrhythmias, entrainment was withheld at this point. The decision a priori was made to proceed with left atrium mapping and inspection of the pulmonary veins to ensure isolation.

**Figure 3:**
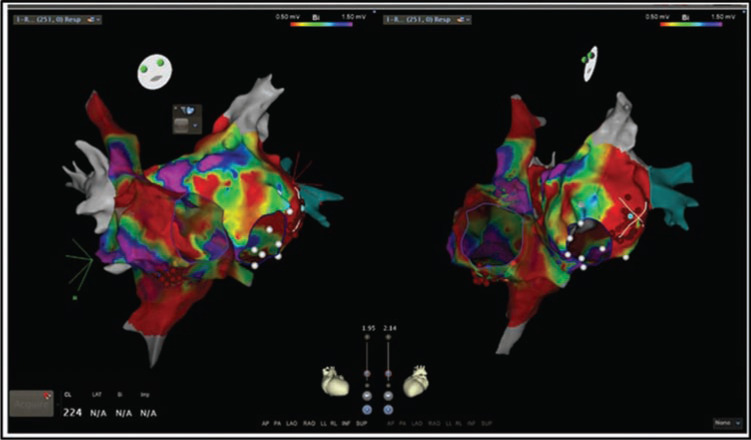
Bi-atrial voltage map. Note the random distribution of low voltage tissue (scale 0.5–1.5 mV). Dense scaring is extensively discernible in both chambers.

**Figure 4:**
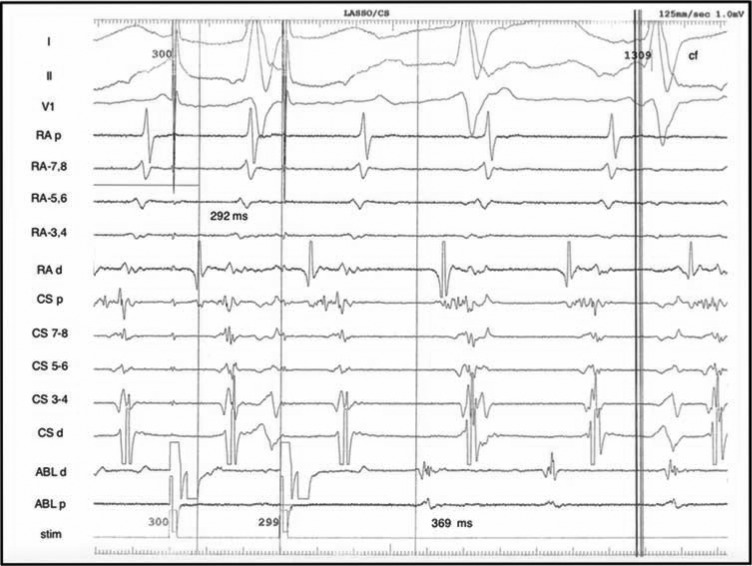
Revealed concealed entrainment when pacing from a catheter located at the CTI.

**Figure 5:**
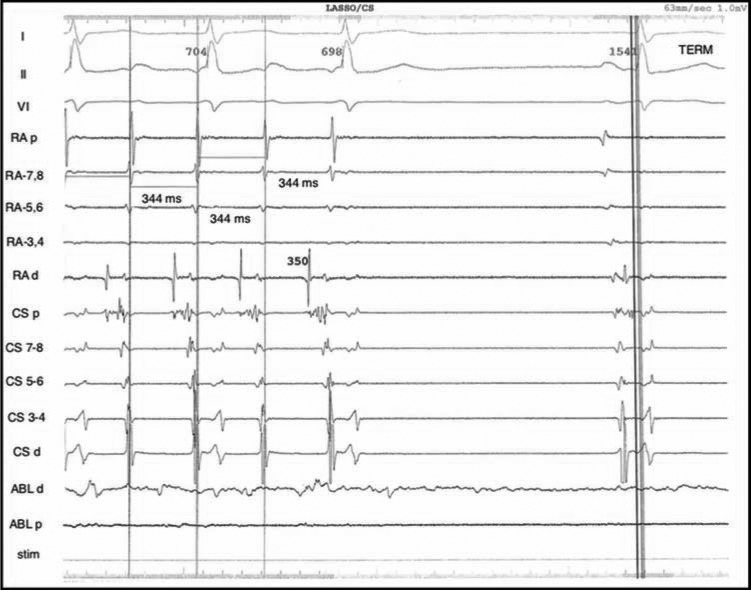
With radiofrequency ablation, the atrial flutter terminates.

Left atrial activation and substrate mapping was carried out. Extensive left atrial scarring was observed; all four pulmonary veins were electrically silent **(Figure 3)**. Based on the degree of clinical suspicion, a test ablation was done at the mitral isthmus, but did not terminate the arrhythmia. Therefore, entrainment was now completed, and the earliest activation was observed at the CTI. Isochronal mapping of the right atrium confirmed CTI-dependent tachyarrhythmia, indicative of AFL **(Figure 4)**. A lesion from the tricuspid valve annulus to the mid-isthmus subsequently terminated the arrhythmia **(Figure 5)**. Ablation was completed with a connecting lesion to the inferior vena cava, and bidirectional block was confirmed prior to the conclusion of the procedure. The patient was not started on antiarrhythmic medications following the procedure.

At the patient’s two-month follow-up appointment, he appeared to be in sinus rhythm, and it was determined that further treatment was not required.

## Discussion

Reduced conduction velocity through scarred myocardium provides the substrate for the development and maintenance of post-surgical arrhythmias. In addition, incomplete lesions, gaps between the pulmonary vein surgical scars, and anatomic barriers following the CMP support the propagation and maintenance of an arrhythmic circuit.^6^

The strongest preoperative predictors of the development of recurrent ATA following the CMP are patient age and left atrial size.^1^ Younger individuals are at greater risk of developing recurrent ATA than those patients who are older (p = 0.0093).^7^ Increases in left atrial diameter were also associated with a greater risk of recurrence, with an odds ratio of 1.42 (p = 0.027).^7^

It should be noted that atrial volume reduction surgery is performed at operators’ discretion in some centers, as literature regarding the efficacy of such procedures in the treatment of AF is scant.

Localization of the reentrant circuit is a significant challenge during catheter ablation in patients with post-CMP ATA.

The surface ECG cannot be used to guide the origin of tachycardia, even though the P-waves were positive in V1 and negative in AVL. According to Akar et al., out of nine patients with recurrent AFL, only one subject had surface ECG consistent with the underlying arrhythmia.^6^ Moreover, the electrocardiographic pattern of this patient was not suggestive of a typical CTI-dependent flutter mechanism.

A recent multicenter study found that 71% of recurrent ATAs were left-sided, which is consistent with previous reports.^4^ It is postulated that extensive scar burden and myocardial remodeling potentiates arrhythmogenesis at the sites of greatest surgical manipulation, which is predominantly in the left atrium during the CMP. Ishii et al. reported that only 14% of recurrent ATAs were due to AFL, while Trumello et al. reported that CTI-dependent flutter occurred in less than 8% of cases studied.^1,8^

Furthermore, incomplete or non-transmural radiofrequency lesions are pro-arrhythmic and predispose the patient to reentrant circuits. In the single-center studies conducted by Chun et al. and Ouyang et al., respectively, restored pulmonary vein conduction and failure to sufficiently isolate the pulmonary veins were the primary sources of recurrent ATA.^2,5^ A study by Akar et al. found that AFL occurred at sites with prior surgical radiofrequency lesions.^6^ Currently, there is no protocol to ensure transmural lesions intraoperatively.

The study by Gopinathannair et al., which was the first multicenter study aimed at defining the characteristics of post-CMP ATA, found that most reentrant circuits following CMP involved the mitral isthmus.^4^ Furthermore, 14 of the 19 patients with mitral isthmus-dependent (MID) flutter had previously undergone ablation at this site during surgery. The technically challenging task of mitral isthmus ablation may lead to incomplete ablation and consequent development of a macro reentrant circuit. Of the 19 patients with MID flutter, 14 had a mitral isthmus line during surgery and ablation at this site.^4^

The decision to investigate the left atrium in this case was made based on the significant clinical suspicion, including surface ECG, intracardiac activation sequence and literature reports of recurrent ATA following CMP. Gopinatthannair et al. reported that 93% of patients had a reconnection of greater than one pulmonary vein following CMP.^4^ The pulmonary veins in this case were silent. Ablation of the mitral isthmus was carried out, as multiple studies have reported predominance of reentry at this site. Mitral isthmus ablation did not terminate the arrhythmia in this case. Owing to remodeling, the electrical pathways in the post-CMP myocardium are altered and therefore are not reliable indicators of location.

One factor, which may be considered as an early indicator of location, is cycle length. Akar et al. reported that the cycle length for the left atrial flutter circuit is significantly shorter at 253 ± 39 ms, as compared with the right atrial flutter circuit, which was 332 ± 63 ms (p < 0.05).^6^ The cycle length for the right atrial flutter circuit in this case was 334 ms, which is consistent with the literature. It may be posited that cycle length could be used to direct ablation procedures following CMP.

## Conclusions

In patients who have undergone extensive left atrial ablation and volume reduction surgery, localization of AFL is significantly difficult. AFL following left atrial surgery is often thought to arise from the conduction slowing resulting from surgical incision-related scar tissue. This case reveals a persistent ATA in the right atrium despite the performance of a left atrial surgical intervention, illustrating the value of a systematic approach of mapping and entrainment. Right atrial mapping should be considered in all cases of possible left AFL before considering ablation, especially when the chamber of interest does not reveal adequate metrics for the critical isthmus.
